# Complete mitogenome of the biocontroller fungus *Purpureocillium* sp. (Ascomycota, Ophiocordycipitaceae, Hypocreales)

**DOI:** 10.1080/23802359.2018.1522982

**Published:** 2018-10-27

**Authors:** Nadya L. Cardona, Nicolás D. Franco-Sierra, Javier Correa Alvarez

**Affiliations:** a Grupo FITOBIOL, Instituto de Biología, Universidad de Antioquia, Medellín, Colombia;; b Grupo CIBIOP, Departamento de Ciencias Biológicas, Universidad EAFIT, Medellín, Colombia;; c Grupo BEC, Departamento de Ciencias Biológicas, Universidad EAFIT, Medellín, Colombia

**Keywords:** *Purpureocillium* sp. fungal biocontrol agent, nematophagous fungus, Whole mitogenome, symphylans biocontrol

## Abstract

The strain *Purpureocillium* sp. UdeA0106 is an antagonist of nematodes, fungi, and garden symphylans from crops with high economic importance in Colombia (Salazar 2013; Salazar et al. 2014; Cardona et al. 2014; Gallego et al. 2014) and is being studied to be proposed as new species. It was included on the 1000 fungal genomes project to elucidate its phylogenetic relationships with other fungi. Purpureocillium’s mitogenome has 23,495 bp of circular size. It contains 15 protein-coding genes without duplications (PCGs), corresponding to the 60% of its total length, 23 transfer genes (7.6% tRNA), two of them duplicated (trnR and trnM), and two ribosomal genes (17.6% rRNA) and a GC content of 28.44%. A phylogenetic tree was proposed using their 14 PCGs mitochondrial genes and was compared with other fungi of the Subphylum Pezizomycotina. Phylogenetics relationships showed UdeA0106 to be close to *P. chlamydosporia* and *M. anisopliae* forming a cluster with other fungal biocontrol agents and separated the strain of plant pathogenic fungi.

*Purpureocillium* sp. UdeA0106 belonging to the order Hypocreales, family Ophiocordycipitaceae, was isolated from Vegas de la Clara land, the property of the University of Antioquia (Gómez Plata, Antioquia, Colombia Lat: 6°34′53.37″N Len: 75°11′43.28″O). UdeA0106 was sequenced into the 1000 fungal genomes project, with the Department of Energy Joint Genome Institute (DOE Joint Genome Institute) support, NCBI ID: 334661 Bioproject PRJNA334661 and was deposited at Universidad de los Andes (Colombia) with the code ANDES-F 1079 and CR-SiB no.15928857199. Genomic tests are being carried out to propose as new species. In contrast, this strain has previously been registered with a high nematicidal capacity against *Meloidogyne* spp. in Colombian flower crops, being able to reduce the number of roots nodulations and controlled the genus *Paratylenchus* spp., showing an increment of the ‘weight productivity’ variable, a desirable aspect in flowers crops (Sánchez and Cardona 2018). Mitogenome’s assembled sequence was obtained from the Joint Genome Institute website (JGI 2017). Protein-coding gene prediction (PCGs), rRNA genes, and tRNA genes were performed with MITOS webserver and ARTEMIS program and then curated manually. To determine the sequences homology with other fungal genus NCBI blast program was used with the default parameters. Phylogenetic analyses were performed using the 14 mitochondrial genes of a database constructed with taxa belonged to the Subphylum Pezizomycotina (Lin et al. 2015).

Phylogenetic reconstruction by BI and ML methods was performed using the automatized PhyPipe workflow (Franco et al. 2016). PhyPipe comprehends; DNA sequences alignment with MAFFT 7.222, partition analysis with PartitionFinder and phylogenetic reconstruction with MrBayes 3.2.6 and Garli 2.01. MrBayes was executed with two MCMC independent races, four chains, 1,000,000 generations, 35% of the ‘burning relative frequency’ and sampling of 100. For the ML analysis, Garli was executed first by doing ML search (five independent searches), then 1000 bootstraps pseudoreplicates were made and the best ML topology was mapped using SumTrees from the DendroPy 4.1.0 program (Sukumaran et al. 2010).

*Purpureocillium* sp. UdeA0106 mitogenome has a weight of 23.495 bp with a circular shape. It contains 15 coding genes with no repetitions (PCGs), corresponding to the 60% of its total size; it also has 23 transference genes (7.6% tRNA), two of which are duplicated (trnR and trnM) and two ribosomal genes (17.6% rRNA). It presents an intronic protein inserted on the rnl gen (Rps3) with 1312 nucleotides. G:C content is 28.44% with a nucleotide composition corresponding to 36.1% of T, 12.9% of C, 33.5% of A, and 15.6% of G. The start and stop codons, ATG/TAA-TAG are the most abundant, while it presents 22 different types of anticodons. In Figure 1 the phylogenetic relations are showing a clade inside the Hypocreales, close to *P. chlamydosporia* and *M. anisopliae* with a boostrap of 100%, close to *H. jecorina* (78%), and clearly separated from those plant pathogenic fungi clades like *Fusarium* spp., and *Gibberella moniliforme*, and others fungal biocontrollers like *B. bassiana*, *B. pseudobassiana*, *C. brongniartii* and *L. muscarinum*. In conclusion, the information obtained, let us to continue to understanding a promissory native biocontrol fungus.

**Figure 1.**
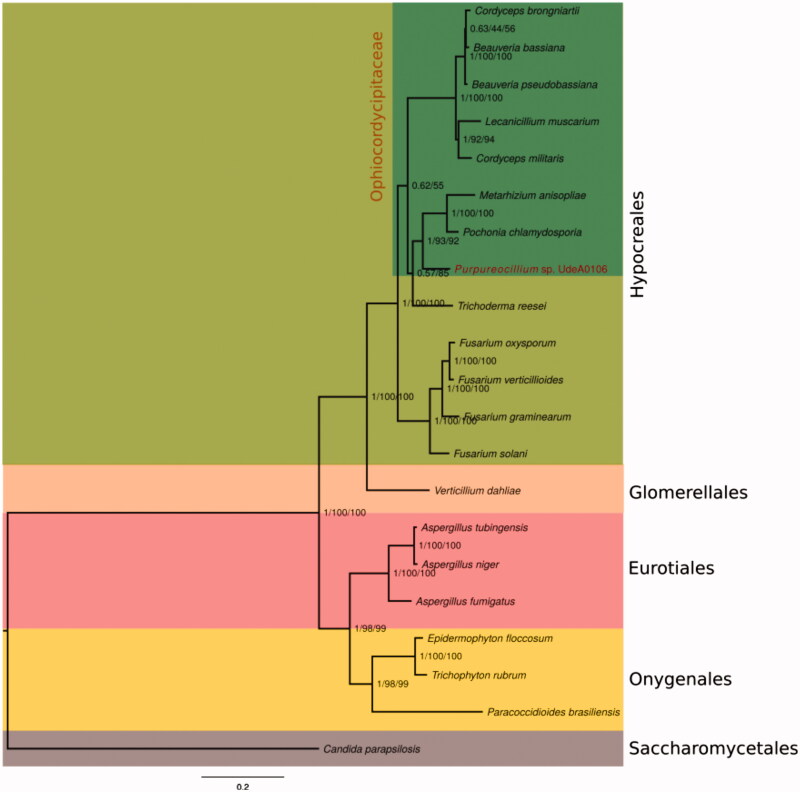
The UdeA0106 phylogenetic analysis of its 14 mitochondrial genes through ML and BI. Internal supports belong to posterior probability values and Bootstrap. As an external group, *C. parapsilopsis* was used. Accession numbers of fungi: *P. chlamydosporia* KF479445, *M. anisopliae* AY884128, *B. bassiana* NC_022708, *C. brongniartii* NC_011194, *L. muscarinum* AF487277*, C. militaris* NC_022834, *H. jecorina* AF447590, *F. solani* NC_016680, *F. graminearum* NC_009493, *F. verticilloides* NC_016687, *F. oxysporum* AY945289, *V. dahliae* DQ351941, *A. fumigatus* JQ346809, *A. niger* NC_007445, *A. tubingensis* NC_007597, *T. rubrum* NC_012824, *E. floccosum* NC_007394, *P. brasiliensis* NC_007935, *C. Parapsilopsis* NC_005253.
